# Supplementary material for: Transposon-Directed Insertion-Site Sequencing Reveals Glycolysis Gene gpmA as Part of the H2O2 Defense Mechanisms in Escherichia coli
Source: Antioxidants (Basel). 2022 Oct 18;11(10):2053. doi: 10.3390/antiox11102053 (PMC9598634; doi:10.3390/antiox11102053)
Supplement: Supplementary file 1 [file antioxidants-11-02053-s001.zip › antioxidants-1974735-supplementary.pdf]

Supplementary Table S1. Primers used to validate the gene replacement by the kanamycin cassette from the Keio collection. Gene accession ID from Ecocyc database.

Name	Sequence	Gene accession ID	Reference
gpmA_seq_F	CCGATGCTCTGTTACGTCAAC	EG11699	This study
gpmA_seq_R	GCGAAGAGTATTCCAGCCTG		This study
dksA_seq_F	TCTTCTATGCGTACCAGCCAG	EG10230	This study
dksA_seq_R	TACATTCTGGTCGCGTGCG		This study
rbsR_seq_F	CATTGCCAGAGGCGATTCG	EG10819	This study
rbsR_seq_R	GGAAGGCACAACGACTGTC		This study
rpoS_seq_F	GACAGTGTTAACGACCATTCTCG	EG10510	This study
rpoS_seq_R	GGAACCAGTTCAACACGCTTG		This study
gpmM_seq_F	CTGGCATCGGCTTGCC	EG12296	This study
gpmM_seq_R	TGGCGTAGATGATGGGCC		This study
hfq_seq_F	GCTATCGCAGGCTGAATGTGTAC	EG10438	This study
hfq_seq_R	GGTCAAACAAGCGTATAACCCTC		This study
nhaA_seq_F	GGCGCAAATTCTTCAATAGCTCG	EG10652	This study
nhaA_seq_R	ACGGAACCTTCTTTATAGACATGCC		This study
corA_seq_F	GTTGCTGTTAACACGAACAAATGG	EG11463	This study
corA_seq_R	GTTGCTGTTAACACGAACAAATGG		This study
dps_seq_F	CCACTATTAGTGTGATAGGAACAGCC	EG11415	This study
dps_seq_R	CCACTATTAGTGTGATAGGAACAGCC		This study

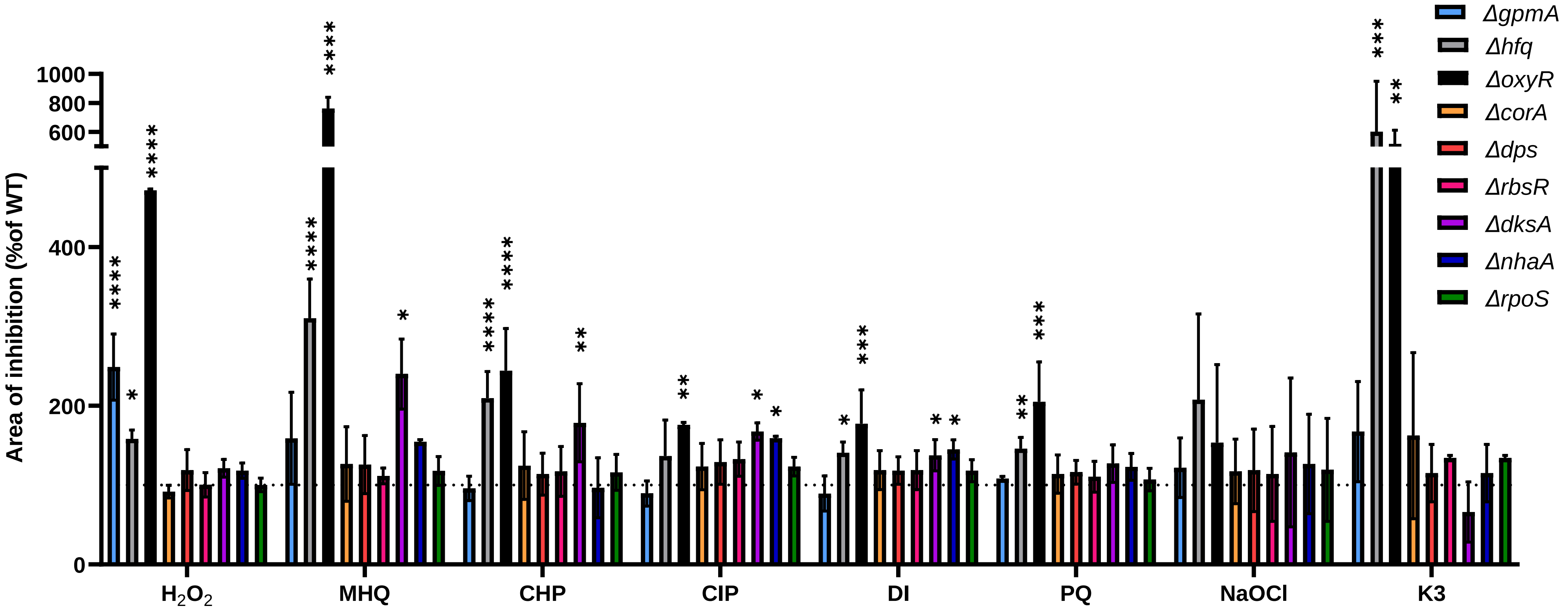

Supplementary Figure S1. Sensitivity of the deletion mutants of the TraDIS exposed to various oxidants. Quantification of the inhibition area normalized to WT for each oxidant and each gene deletion. Oxidant applied on each disk (CHP: cumene hydroperoxide, MHQ: methylhydroquinone, H_2O_2: hydrogen peroxide, CIP: ciprofloxacin, Di: diamide, AMP: amplicillin, K3: menadione, NaOCL: sodium hypochlorite, DMSO: dimethyl-sulfoxide); One-way ANOVA with Tukey multiple comparison was performed separately for each oxidant on the area of inhibition of the WT, *ΔkatG* and the 9 mutants identified by TraDIS. The significance of the difference with the WT is represented on the normalized data by stars where */**/***/**** correspond to $p < 0.05$, 0.01, 0.001, 0.0001 respectively (mean +/- SD, N=3).

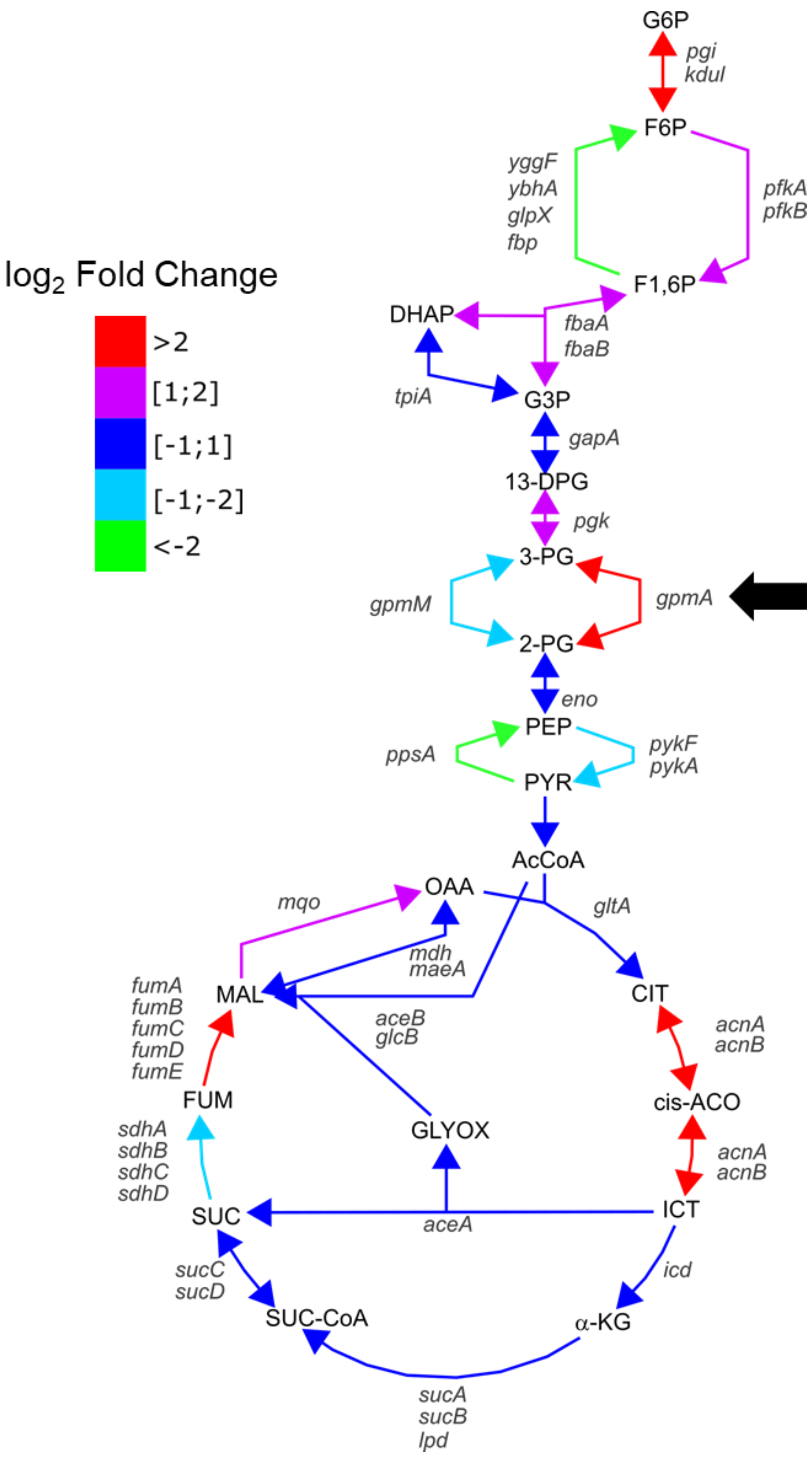

Supplementary Figure S2. Schematic diagram of H_2O_2-induced transcriptional changes of glycolysis and TCA cycle. The color code describes the differential expression of the genes coding for the enzyme catalyzing the described reactions 10 minutes after exposition to 2.5 mM H_2O_2 compared to no treatment. The *gpmA* gene is indicated by a black arrow. Data from previously performed RNA-seq (deposited on ENA with the accession number: PRJEB51098).

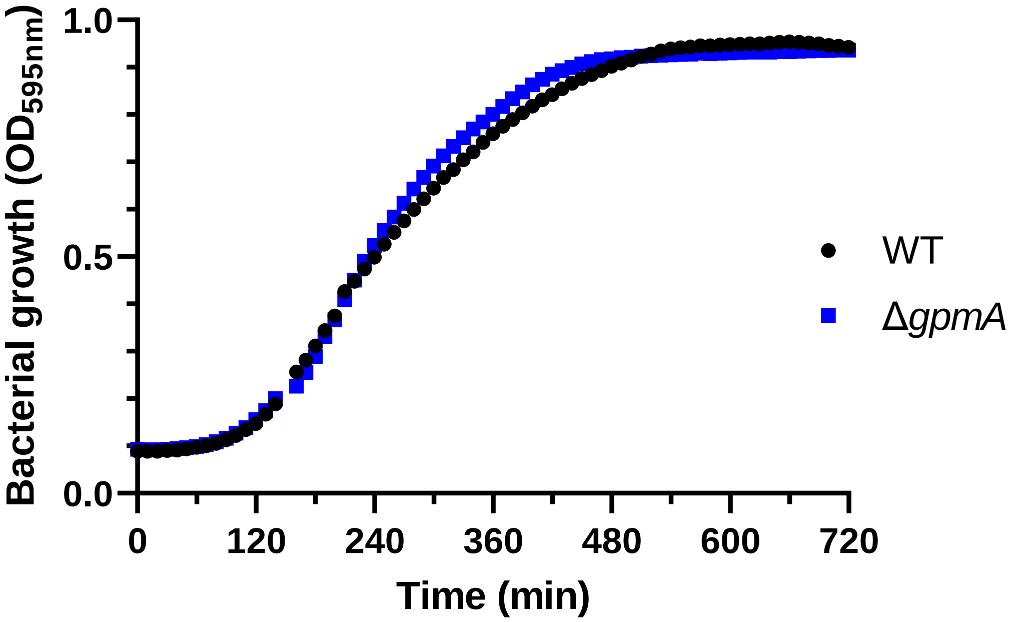

Supplementary Figure S3. The deletion of *gpmA* did not affect bacterial growth. Growth curves of the WT and the *ΔgpmA* mutant in liquid LB over time (mean, *N*=3).

An overnight culture of E. coli was normalized to 1.0 Mc Farland using a Densimat (bioMérieux) and further diluted 1:10 in fresh LB. This bacterial culture was grown in a volume of 1 mL in a 24-well plate (142475, ThermoFisher) and incubated at 37 °C with 5 mm orbital shaking in an Infinite 200PRO plate reader (Tecan). Absorbance was measured every 10 min at an optical density of 595 nm.
